# Discovery of a novel fluorescent chemical probe suitable for evaluation of neuropilin‐1 binding of small molecules

**DOI:** 10.1002/ddr.21641

**Published:** 2020-01-20

**Authors:** Daniel Conole, Yi‐Tai Chou, Anastasia Patsiarika, Valery Nwabo, Eleni Dimitriou, Christelle Soudy, Filipa Mota, Snezana Djordjevic, David L. Selwood

**Affiliations:** ^1^ Wolfson Institute for Biomedical Research University College London London UK; ^2^ Institute of Structural and Molecular Biology University College London London UK

**Keywords:** fluorescence polarization, neuropilin‐1, surface plasmon resonance

## Abstract

Neuropilin‐1 (NRP1) is emerging as an important molecule in immune signaling where it has been shown to modulate the actions of TGF‐β1 in macrophages and regulatory T cells. The development of cost‐effective and reliable assays for NRP1 binding is therefore important. We synthesized three new NRP1 small molecule fluorophores and examined their performance as fluorescent polarization probes. One molecule DS108 exhibited favorable binding and fluorescent characteristics and allowed us to establish a simple assay suitable for medium to high throughput screening of small molecules.

**Abbreviations**
FP: fluorescence polarization
NRP1: neuropilin‐1
SPR: surface plasmon resonance
TGF‐β1: transforming growth factor‐β1
VEGF: vascular endothelial growth factor

## 1 INTRODUCTION

The ability of neuropilin‐ 1 (NRP1) to bind and augment the action of growth factors such as vascular endothelial growth factor (VEGF), transforming growth factor‐β1 (TGF‐β1), placental growth factor (PLGF), HGF (scatter factor) and Semaphorins 3A, 4F (Pellet‐Many, Frankel, Jia, & Zachary, 2008) are consistent with its emerging role as a tumor promoting receptor acting by a number of mechanisms. These mechanisms can be via modulation of the immune response to tumors through affecting the function of macrophages (Nissen, Selwood, & Tsirka, 2013) and regulatory T cells (T_regs_) (Delgoffe et al., 2013), via angiogenesis by promotion of NRP1/VEGF‐A signaling (Pan et al., 2007); through prevention of tumor cell migration by binding to NRP1 (Jia et al., 2010); or a direct effect on the tumor cells (Grun, Adhikary, & Eckert, 2016). The binding site on NRP1 b1 is formed by loops in the protein structure forming a “receptor” for a C‐terminal arginine residue (Jarvis et al., 2010). This is variously termed in the literature as the tuftsin site, arginine receptor, or aromatic box (Y297, W301, and Y353). The peptide and small molecule antagonists reported dating all bind to this site (Peng, Bai, Zhu, Hu, & Xu, 2019). Many assay systems have been developed to detect NRP1 binding ranging from classical radiolabelled formats (Jia et al., 2006), surface plasmon resonance (SPR), luminescence (Powell et al., 2018) to bead based systems (Huang et al., 2019) and homogeneous time resolved fluorescence (HTRF) (Auriau et al., 2018). These assays may be expensive and complicated to implement. The development of simple and reliable assays for NRP1 binding and function is therefore of importance.

**Figure 1:**
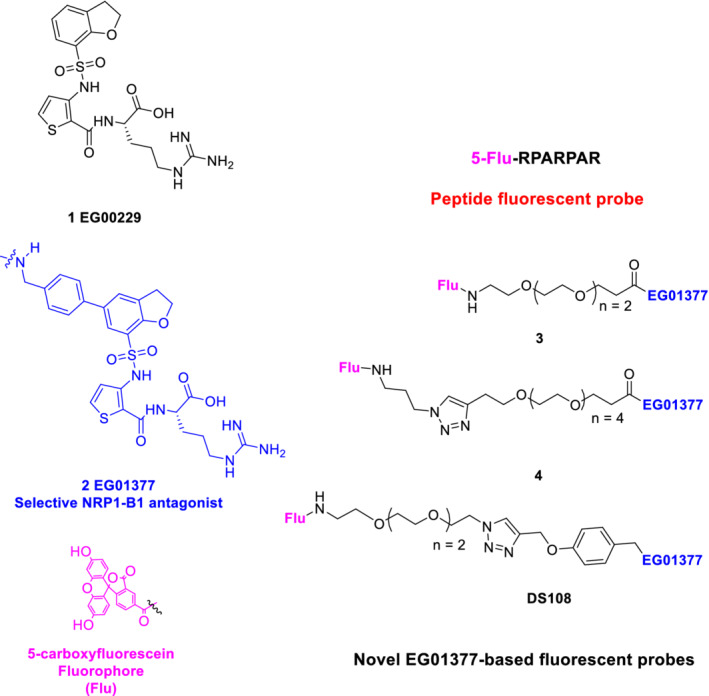
Peptide fluorescein probe and chemical structures of novel NRP1‐B1 fluorescent probes used in this study

Carboxyfluorescein (Flu)—labeled RPARPAR peptide is a known NRP1 ligand probe (National Center for Biotechnology Information, 2019. PubChem BioAssay Database; AID = 602438, https://pubchem.ncbi.nlm.nih.gov/bioassay/602438) for NRP1 fluorescence polarization (FP) experiments (Figure 1). However, while this peptide was reported as suitable for single point binding analysis in our hands it exhibited poor performance in competition experiments. We therefore initiated a study to identify a fluorescent probe suitable for the evaluation of ligand binding to the b1 or b1b2 domains of NRP1 (NRP1‐b1b2) via FP‐based ligand competition experiments.

The new probes were based on the structure of EG01377 **2**, a compound identified in our group and known to be selective for NRP1 over the closely related NRP2 receptor and possess submicromolar NRP1 antagonistic properties (Powell et al., 2018). The design incorporated several combinations of polyethylene glycol (PEG) and triazole click linker units to examine whether the fluorophore could be attached without compromising the intrinsic binding of EG01377 (Figure 1).

Three structurally distinct probes (3, 4, and DS108, Figure 1) were synthesized and evaluated against the literature RPARPAR probe. We used SPR as an orthogonal biophysical technique to assess the binding properties of these probes and to determine their respective K_D_ values. From this, we were able to quickly identify conditions in which to assess the FP dynamic range of each probe, by optimizing probe and NRP1‐b1 concentrations. Subsequent NRP1‐b1 protein titration experiments and competition assays with known NRP‐b1 ligands (EG01377 and EG03286) confirmed the utility of this assay for the medium‐to‐high throughput discovery of novel ligands for NRP1‐b1 binding.

## 2 METHODS

### 2.1 SPR

**Scheme 1:**
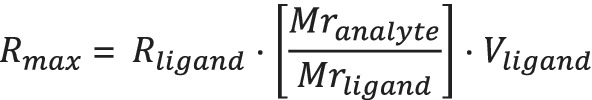
Theoretical R_max_ equation, where R_ligand_ = amount of protein loaded in the SPR chip in response units; Mr_analyte_ = molecular weight of the compound of interest; Mr_ligand_ = molecular weight of the immobilized protein; V_ligand_ = stoichiometry of the binding interaction between the ligand and the analyte

All SPR analysis was performed on a BIAcore T200 system using series S CM5 sensor chips. The Biotage SPR is effectively a stop‐flow instrument, and dissociation from the immobilized protein is initiated by the absence of analyte (ligand) when buffer alone is perfused, this setup allows the determination of on and off rates and equilibrium binding constants. Relatively high DMSO concentrations are normal for SPR experiments to limit solubility problems and minimize nonspecific aggregation. Extensive DMSO controls are included and automatically subtracted from the sensorgrams. Sensorgrams were double referenced by subtracting the response on a reference flow cell and a blank sample. Ligands were evaluated against the NRP1‐b1 domain. NRP1‐b1 was covalently attached to a CM5 chip via amine coupling (Powell et al., 2018) with a surface density of 2,000–3,000 RU. Binding of novel fluorescent probes (0.4–100 μM) were analyzed by multicycle sequential injections (30–120 s association time) followed by undisturbed dissociation (30–60 s). A regeneration step was not used. Peptide stocks were dissolved in dimethyl sulfoxide (DMSO), and the final sample solutions for kinetic affinity experiments contained 3% DMSO in 1× phosphate‐buffered saline P20 buffer (PBS‐P, Cat no 28995084, GE Healthcare Ltd.). DMSO solvent effects were corrected for with eight calibration solutions (0.5–1.8% DMSO in PBS‐P). Equilibrium constants (K_D_) were calculated using either kinetic or affinity models, assuming simple 1:1 (Langmuir) binding. Data processing and analysis were performed using BIAevaluation and OriginPro software. The theoretical R_max_ (the maximal feasible signal between a ligand—analyte pair) for each compound/protein pair was calculated using Scheme 1 (Marquart, 2017).

The experimentally observed R_max_ was then calculated as a percentage of the theoretical R_max_ as a quality control measure.

### 2.2 FP

Initial experiments were performed using PBS‐P buffer with 3% DMSO in a final volume of 80 μl. The reaction plates or tubes were kept on ice during pipetting.

Saturation binding: dynamic range and protein titration experiments were performed at probe concentrations selected with guidance from in‐house SPR analysis, literature K_D_ of the untagged compound, and an FP technical resource guide (Invitrogen, 2006). The NRP‐b1 concentrations ranged from 10 nM to 30 μM. Samples were prepared in the following order—NRP1‐b1 protein (serial dilution as shown in Figure X, 40 μl) in PBS‐P buffer, and FP probes (concentrations shown in Tables 2, 40 μl) in PBS‐P buffer.

Competition experiments: FP samples were prepared in the following order—ligand in PBS‐P buffer (20 μl), NRP1‐b1 protein (1,400 nM, 20 μl) in PBS‐P buffer, and DS108 probe (1,500 nM, 40 μl) in PBS‐P buffer. FP was measured and normalized to experiment controls (buffer + probe, buffer + probe + protein) using a BMG Labtech PHERAstar® plate reader (filter settings: 485 nm [excitation] and 520 nm [emission]). Background FP was blanked using a PBS‐P buffer only control.

#### 2.2.1 Final optimized protocol for FP

The final FP assays were realized at 50 nM of DS108, 300 nM of NRP1 b1b2 All solutions contained a buffer constituted of 10 mM HEPES and 0.5% DMSO, reflecting the low initial concentration of the molecule under investigation needed for the assay. EG00229 and EG01377 starting concentrations were 5 μM only (stock solution consisted of 10 mM of the compounds diluted in 100% DMSO). DMSO derived from DS108 (stock solution 10 mM in 100% DMSO) is at a very low concentration; 100 times lower compared to the 0.5% derived from the compounds and did not interfere with the acquisition of the competition assay curves. The final sample volume in each well was 30 μl. Data were analyzed using OriginPro software. The assays were performed using PHERAstar®. From the general settings of the PHERAstar® fluorescence polarization software the Settling time was changed from 0.3 to 0.5 s, which is important in order to enhance the assay's accuracy at lower probe concentrations.

**Scheme 2:**
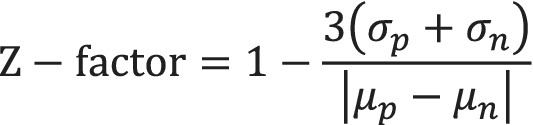
Calculation of Z‐factor

Data processing: raw data were processed using OriginPro curve fitting software to obtain the IC_50_s and error values shown. A dose–response curve fitting model was utilized for the competition experiments. A web based IC_50_‐to‐K_i_ converter that computes K_i_ values from experimentally determined IC_50_ values was employed (Cer, Mudunuri, Stephens, & Lebeda, 2009) (https://bioinfo-abcc.ncifcrf.gov/IC50_Ki_Converter/index.php) Each experiment was conducted in triplicate and repeated two times. The statistical reproducibility of this assay was evaluated using the z factor equation (Scheme 2).

## 3 CHEMISTRY

All starting materials were from commercial sources or synthesized by literature procedures as indicated. FAM‐RPARPAR was purchased from Peptide Protein Research Ltd, Hampshire, UK.

### 3.1 Fluorescent probe 3: (S)‐5‐((1‐(4‐(7‐(N‐(2‐((1‐carboxy‐4‐guanidinobutyl)carbamoyl)thiophen‐3‐yl)sulfamoyl)‐2,3‐dihydrobenzofuran‐5‐yl)phenyl)‐3‐oxo‐6,9,12‐trioxa‐2‐azatetradecan‐14‐yl)carbamoyl)‐2‐(6‐hydroxy‐3‐oxo‐3H‐xanthen‐9‐yl)benzoic acid

#### 3.1.1 Stage 1

1377‐PEG‐amine. (3‐((5‐(4‐(14‐amino‐3‐oxo‐6,9,12‐trioxa‐2‐azatetradecyl)phenyl)‐2,3‐dihydrobenzofuran)‐7‐sulfonamido)thiophene‐2‐carbonyl)‐L‐arginine.

To the Fmoc‐PEG‐acid (44.3 mg, 0.10 mmol, 1.0 eq) in DMF was added DMAP (36.7 mg, 0.3 mmol, 3.0 eq) and PYBOP (52.0 mg, 0.1 mmol, 1.0 eq). The reaction was then stirred for 10 min. The 1,377 di TFA salt (83.1 mg, 0.1 mmol, 1.0 eq) was added in portions. LCMS showed evidence for product but with partial loss of the Fmoc group. The DMF was removed on the rotary evaporator (~1 mmHg) when LCMS indicated Fmoc loss complete. TFA (50 μl) was added to the residue with ACN/H_2_O 50/50 (3 ml). This was applied directly to the reverse phase column, eluting with a gradient of 5–95% ACN/H_2_O 0.1% TFA and the product isolated as the TFA salt and freeze‐dried to give a solid (37.5 mg, 0.041 mmol, 41.0%).

^1^H NMR (600 MHz, Acetonitrile‐*d*_3_) δ 7.73–7.66 (m, 2H), 7.54–7.47 (m, 2H), 7.44 (d, *J* = 5.5 Hz, 1H), 7.35 (d, *J* = 8.1 Hz, 2H), 7.30 (d, *J* = 5.5 Hz, 1H), 4.68 (td, *J* = 8.8, 1.6 Hz, 2H), 4.52 (dd, *J* = 9.5, 4.9 Hz, 1H), 4.40 (s, 2H), 3.73 (t, *J* = 5.8 Hz, 2H), 3.66–3.62 (m, 2H), 3.61–3.56 (m, 8H), 3.25 (t, *J* = 8.8 Hz, 2H), 3.15 (hept, *J* = 6.9 Hz, 2H), 3.02 (t, *J* = 5.0 Hz, 2H), 2.52 (t, *J* = 5.8 Hz, 2H), 1.83 (ddt, *J* = 13.8, 9.5, 7.5 Hz, 1H), 1.69–1.62 (m, 2H).

^13^C NMR (151 MHz, CD_3_CN) δ 164.84, 158.05, 157.45, 143.21, 139.07, 139.00, 134.10, 132.93, 130.92, 130.35, 129.95, 128.79, 128.03, 127.73, 126.05, 121.74, 121.32, 114.13, 74.85, 71.04, 70.84, 70.54, 70.25, 67.71, 67.15, 52.94, 43.39, 41.69, 40.40, 36.90, 29.46, 28.72, 25.67.

#### 3.1.2 Stage 2

To the amine TFA salt (24.6 mg, 24.166 μmol, 1.000 eq) in DMF was added 5‐carboxyfluorescein (9.1 mg, 24.166 μmol, 1.0 eq), DMAP (11.7 mg, 95.8 μmol, 3.96 eq) and PYBOP (12.0 mg, 23.059 μmol, 0.9 eq) stirred overnight then water (0.5 ml) added and the residue applied directly to a reverse phase (C18) column and eluted with ACN/H_2_O (5–95%) containing 0.1% TFA. Fractions containing product were concentrated on a rotary evaporator and freeze‐dried. Product isolated as a yellow solid (25.6 mg, 20.281 μmol, 83.9%). LCMS ESI [M + H]^+^ 1,148.

### 3.2 Fluorescent probe 4: (3‐((5‐(4‐(1‐(1‐(3‐(3′,6′‐dihydroxy‐3‐oxo‐3H‐spiro(isobenzofuran‐1,9′‐xanthene)‐5‐carboxamido)propyl)‐1H‐1,2,3‐triazol‐4‐yl)‐17‐oxo‐2,5,8,11,14‐pentaoxa‐18‐azanonadecan‐19‐yl)phenyl)‐2,3‐dihydrobenzofuran)‐7‐sulfonamido)thiophene‐2‐carbonyl)‐L‐arginine

#### 3.2.1 Stage 1

(3‐((5‐(4‐(3‐oxo‐6,9,12,15,18‐pentaoxa‐2‐azahenicos‐20‐yn‐1‐yl)phenyl)‐2,3‐dihydrobenzofuran)‐7‐sulfonamido)thiophene‐2‐carbonyl)‐L‐arginine.

To a stirred solution of **EG01377 2** (0.026 g, 0.045 mmol, 1.2 eq.) in DMF (2 ml), was added triethylamine (0.02 ml, 0.148 mmol, 4 eq.). A solution of acetylene‐PEG_4_‐NHS ester (0.015 g, 0.037 mmol 1 eq.) in DMF (1.5 ml) was then added, and left stirring overnight at RT. The crude was concentrated for reverse phase LC purification, with 0:100–85:15 methanol: water (0.1% formic acid) gradient elution, to give **5** as a colorless oil (0.03 g, 0.03 mmol, 95.0% yield). ^**1**^ **H NMR** (600 MHz, DMSO‐*d*_6_) δ 1.63–1.77 (m, 3H, 25), 2.39 (t, *J* = 6.5 Hz, 1H, 8), 3.45–3.56 (m, 16H, 12, 13, 15, 16, 18, 19, 21, 22), 3.64 (t, *J* = 6.4 Hz, 2H, 24), 4.13 (d, *J* = 2.4 Hz, 2H), 4.23 (s, 1H), 4.29 (d, *J* = 5.9 Hz, 2H, 28), 4.59 (dq, *J* = 8.8, 13.9 Hz, 2H), 6.87 (s, 2H, 38), 7.16 (d, *J* = 5.4 Hz, 1H, 30), 7.23 (d, *J* = 5.4 Hz, 1H, 6), 7.28–7.32 (m, 2H, 5, 31), 7.46–7.49 (m, 2H, 4, 27), 7.53 (dd, *J* = 1.1, 2.1 Hz, 1H, 52), 7.68 (d, *J* = 2.0 Hz, 1H, 51), 8.22 (s, 1H, 34), 8.39 (t, *J* = 5.9 Hz, 1H), 10.09 (s, 1H, 54).^**13**^ **C NMR** (151 MHz, DMSO‐*d*_6_) δ 39.13, 39.28, 39.28, 40.02, 40.22, 68.52, 69.60, 69.77, 69.82, 77.17. **LCMS**: MS *m/z* 874.4 [M + H]^+^.

#### 3.2.2 Stage 2

To a stirred solution of N‐(3‐azidopropyl)‐3′,6′‐dihydroxy‐3‐oxo‐3H‐spiro[isobenzofuran‐1,9′‐xanthene]‐5‐carboxamide (23.6 mg, 0.027 mmol 1.0 eq.) and EG01377‐PEG_4_‐acetylene **5** (12.4 mg, 0.027 mmol 1.0 eq.) in 2.0 ml of ^*t*^BuOH/water (1:1) solution was added sodium ascorbate (50 mg, 0.27 mmol 10.0 eq.) dissolved in 0.5 ml of ^*t*^BuOH/water (1:1), followed by aqueous copper (II) sulfate pentahydrate (33.7 mg, 0.135 mmol 5.0 eq.) dissolved in 0.5 ml of ^*t*^BuOH/water (1:1). The mixture was left stirring at RT for 2 hr, after which it turned to red‐orange mixture and the solid product precipitated. The solvent was evaporated and 5 ml of aqueous solution of tris(3‐hydroxypropyltriazolylmethyl)‐amine (THPTA) (58.7 mg, 0.135 mmol 5.0 eq.) was added to the dried solid. The solution was filtered through IST Phase separator frit with a layer of celite, and the solid was washed with 5 ml of water to remove excess copper. Finally, DMF wash diluted the red solid layer of product, which was then obtained after solvent evaporation (5.1 mg, 0.004 mmol, 14.2% yield). The solid was ~85% pure by LCMS ESI 1331 [M + H]^+^.

### 3.3 Fluorescent probe DS108: (3‐((5‐(4‐(1‐(1‐(3‐(3′,6′‐dihydroxy‐3‐oxo‐3H‐spiro(isobenzofuran‐1,9′‐xanthene)‐5‐carboxamido)propyl)‐1H‐1,2,3‐triazol‐4‐yl)‐17‐oxo‐2,5,8,11,14‐pentaoxa‐18‐azanonadecan‐19‐yl)phenyl)‐2,3‐dihydrobenzofuran)‐7‐sulfonamido)thiophene‐2‐carbonyl)‐L‐arginine

#### 3.3.1 Stage 1

(3‐((5‐(4‐(((4‐(prop‐2‐yn‐1‐yloxy)benzyl)amino)methyl)phenyl)‐2,3‐ dihydro benzofuran)‐7‐sulfonamido)thiophene‐2‐carbonyl)‐*L*‐arginine.

To the 4‐(prop‐2‐yn‐1‐yloxy)benzaldehyde (Enamine, EN300‐56436) (16 mg, 0.1 mmol) in DMF (1.6 ml) was added EG01377 di‐TFA salt (162 mg, 0.2 mmol), and acetic acid (30 μl) and the reaction stirred at 50° for 1 hr. Then sodium triacetoxyborohydride (80 mg, 0.4 mmol) was added and the reaction stirred at 50° overnight. Water (1 ml) was added to the reaction mixture which was applied directly to a reverse phase column and eluted with ACN/H_2_O (5–95%) containing 0.1% TFA. The product was isolated as a solid. Yield 14.5 mg, (0.020 mmol, 19.9%). LCMS ESI [M + H]^+^ 731.

^1^H NMR (600 MHz, Methanol‐*d*_4_) δ 7.63 (dd, *J* = 15.3, 1.8 Hz, 2H), 7.53 (d, *J* = 7.4 Hz, 2H), 7.44 (d, *J* = 8.2 Hz, 2H), 7.37 (d, *J* = 5.5 Hz, 1H), 7.34 (d, *J* = 8.2 Hz, 2H), 7.16 (dd, *J* = 5.5, 2.2 Hz, 1H), 6.98 (d, *J* = 8.6 Hz, 2H), 4.67 (d, *J* = 2.4 Hz, 2H), 4.65–4.58 (m, 2H), 4.52–4.47 (m, 1H), 3.19–3.11 (m, 6H), 2.87 (t, *J* = 2.4 Hz, 1H), 2.00–1.92 (m, 1H), 1.79–1.72 (m, 1H), 1.67–1.57 (m, 2H).

^13^C NMR (151 MHz, Methanol‐*d*_4_) δ 173.30, 163.85, 158.53, 157.06, 156.59, 141.35, 140.52, 132.48, 131.61, 131.02, 130.17, 129.89, 128.47, 126.88, 125.10, 123.41, 120.62, 120.33, 115.05, 77.82, 75.51, 73.47, 55.06, 51.57, 49.98, 49.87, 40.30, 28.13, 26.53, 25.06.

#### 3.3.2 Stage 2

N‐(2‐(2‐(2‐(2‐azidoethoxy]ethoxy)ethoxy)ethyl)‐3′,6′‐dihydroxy‐3‐oxo‐3H‐spiro[isobenzofuran‐1,9′‐xanthene)‐6‐carboxamide.

To 1‐Amino‐11‐azido‐3,6,9‐trioxaundecane (72 mg, 65 μl, 0.33 mmol) in DMF (1 ml) was added 5,6‐carboxyfluorescein (mixture of isomers) (125 mg, 0.33 mmol) followed by PyBOP (173 mg, 0.33 mmol) and DIPEA (84 mg, 113 μl, 0.6 mmol). And the reaction stirred overnight. Water (0.2 ml) was added to the reaction mixture and this applied directly to a reverse phase C18 column eluting with 10 to 90% ACN in water containing 0.1% TFA. The product was isolated as a yellow gum. Yield (74.5 mg, 0.129 mmol, 38.9%). LCMS ESI [M + H]^+^ 576.

^1^H NMR (600 MHz, Acetone‐*d*_6_) δ 8.44 (dd, *J* = 1.6, 0.7 Hz, 0.5H), 8.31 (dd, *J* = 8.0, 1.6 Hz, 0.5H), 8.23 (dd, *J* = 8.0, 1.4 Hz, 0.5H), 8.06 (dd, *J* = 8.0, 0.8 Hz, 0.5H), 7.74 (dd, *J* = 1.4, 0.8 Hz, 0.5H), 7.38 (dd, *J* = 8.0, 0.8 Hz, 0.5H), 6.77 (t, *J* = 2.4 Hz, 2H), 6.70 (d, *J* = 8.7 Hz, 2H), 6.63 (ddd, *J* = 8.7, 2.4, 1.3 Hz, 2H), 3.72–3.67 (m, 1H), 3.66–3.58 (m, 7H), 3.57–3.54 (m, 0.5H), 3.53–3.47 (m, 5H), 3.41–3.30 (m, 2H), 2.08–2.03 (m, 2H).

^13^C NMR (151 MHz, Acetone) δ 168.91, 168.83, 165.95, 165.93, 162.28, 160.38, 160.36, 158.63, 158.36, 156.01, 154.11, 153.36, 153.33, 142.06, 137.63, 135.16, 130.36, 130.26, 130.17, 129.78, 128.17, 125.62, 125.16, 124.14, 123.38, 116.86, 114.97, 113.39, 113.34, 111.22, 111.18, 103.30, 103.28, 79.22, 79.01, 78.79, 71.32, 71.25, 71.18, 71.16, 71.13, 71.11, 70.97, 70.81, 70.70, 70.67, 70.61, 70.10, 69.95, 51.36, 51.32, 51.29, 47.23, 40.63, 40.56.

#### 3.3.3 Stage 3

To a stirred solution of EG01377‐phenoxy‐acetylene (14.9 mg, 0.020 mmol, 1 eq) and the Flu‐PEG‐azide (11.8 mg, 0.020 mmol) in DMF (0.5 ml) was added the copper complex R3 (7.6 mg, 0.02 mmol) premixed with the lutidine R4 (0.1 ml, 0.86 mmol) in DMF (0.5 ml). The reaction was stirred overnight and then water (0.5 ml) added and the mixture applied directly to a reverse phase column eluted with ACN/H_2_O (5–95%) containing 0.1% TFA. The product was isolated as a yellow gum (12.0 mg, 0.009 mmol, 45.0%). LCMS ESI (MH + H)^+^ 1307. HRMS theoretical for [C_65_H_66_N_10_O_16_S_2_ + H]^+^ 1307.4172, measured 1307.4137 Error:−0.19 ppm.

## 4 RESULTS AND DISCUSSION

### 4.1 Chemistry

**Figure 2:**
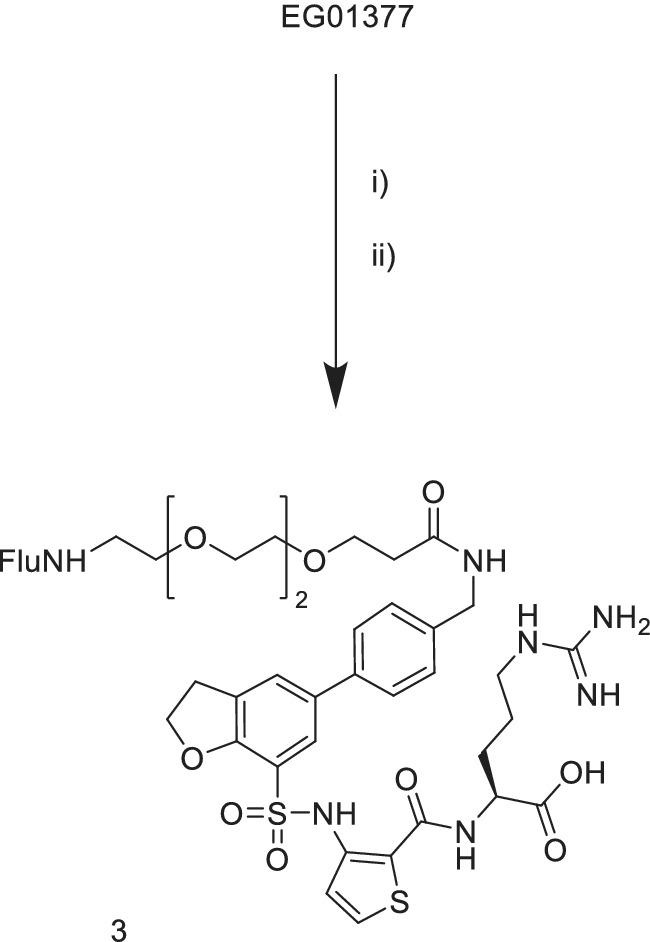
Synthesis of probe (3). Reagents: (i) Fmoc‐PEG2‐acid, DMF, DMAP, PyBOP; (ii) 5‐carboxyfluorescein, DMF, DMAP, PyBOP

**Figure 3:**
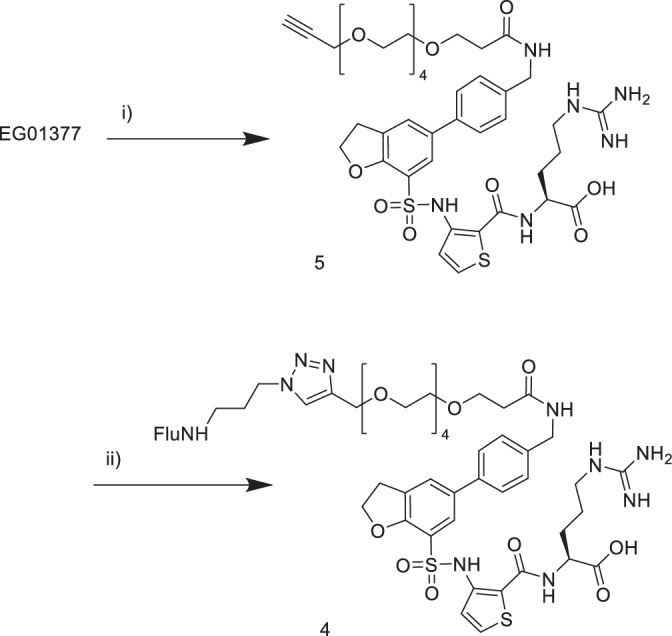
Synthesis of probe (4) (i) AlkynePEG4‐acid‐NHS ester, TEA, DMF, RT overnight, (ii) Flu‐NH(CH_2_)_3_N_3_, CuSO_4_.5H_2_O, sodium ascorbate, ^t^BuOH, H_2_O

**Figure 4:**
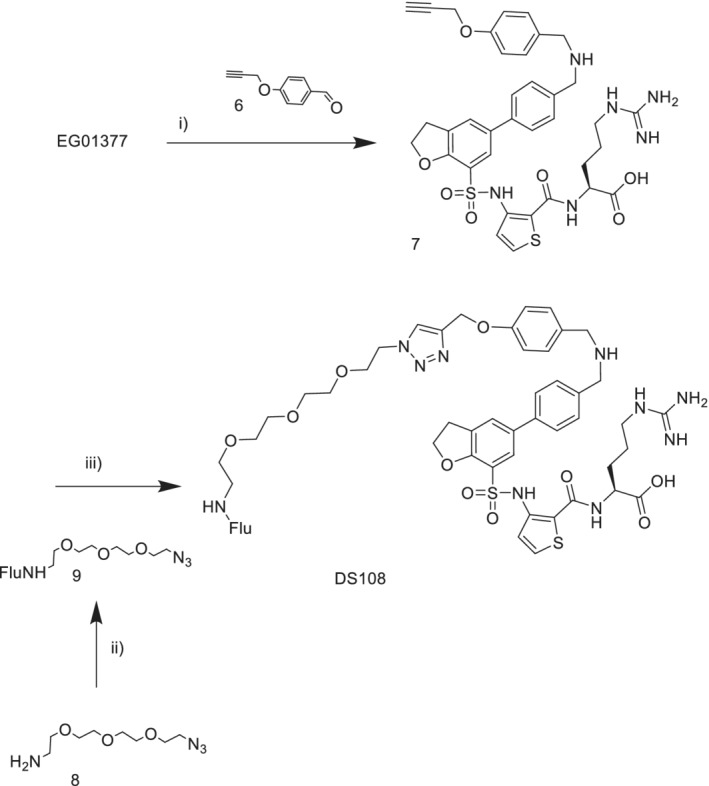
Synthesis of fluorescent probe DS108. (i) NaBH(OAc)_3_, DMF; (ii) Cu(SO_4_)_2_, sodium ascorbate, ^t^BuOH, H_2_O

The synthesis of the EG01377 derived probes was carried out using the unprotected EG01377 as the starting material. This minimized the number of synthetic steps required. Reaction mixtures could be purified directly by reverse phase chromatography. Fluorescent probe **3** was prepared from EG1377 in two steps (Figure 2). First the Fmoc‐PEG‐acid was preactivated with PyBOP in DMF and DIPEA then the EG01377 added. The Fmoc group was removed in the work‐up then the resulting amine coupled with 5,6‐carboxyfluorescein. Probe **4** was synthesized using a similar coupling of alkyne‐PEG‐acid to EG01377 to provide alkyne **5** (Figure 3). This was followed by a copper mediated Huisgen click cycloaddition reaction (Rostovtsev, Green, Fokin, & Sharpless, 2002) with the fluorescein alkyl azide to give the product. For DS108 we wanted to preserve the basic amine group on the EG01377 structure which was known to be important for binding. EG01377 was converted to the phenoxy alkyne **7** by reductive amination with the commercially available aldehyde **6** (Figure 4). Reaction of 5‐carboxyfluorescein with the PEG amino azide **8** provided the fluorescein‐PEG‐azide. Finally, a copper mediated click reaction gave the desired DS108.

### 4.2 SPR and FP

**Figure 5:**
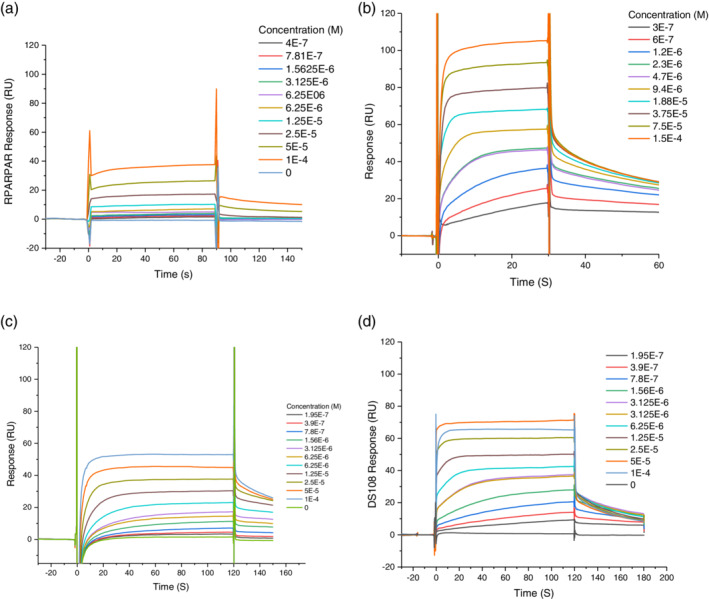
Representative raw SPR sensorgrams for Flu‐tagged chemical probes against NRP1‐B1—**A** RPARPAR; **B** Probe **3**; **C** Probe **4**; **D** DS108

We have previously validated the SPR system for NRP1 and it provides similar data to other assay systems such as biotinylated‐VEGF—luciferase or radiolabelled VEGF. Fluorescently tagged probes RPARPAR, **3**, **4**, and DS108 exhibited SPR K_D_ values of 69.03 ± 22, 24.6 ± 6.3, 3.3 ± 0.2, and 2.13 ± 0.81 μM, respectively (Figure 5, Table 1). This was compared with the positive control EG01377, which possessed a K_D_ = 1.32 ± 0.08 μM (Powell et al., 2018). All probes demonstrated reasonable equilibrium binding characteristics as evidenced by their sensorgrams and calculated R_max_ values as shown in Table 1.

**Table 1:** Summary of SPR and FP binding properties of NRP1‐b1 fluorescent probes

	SPR	
5‐flu probe	K_D_ (μM, affinity)	Theoretical R_max_ (%)
RPARPAR	69.03 ± 22	60.61
**3**	24.6 ± 6.3	77
**4**	3.3 ± 0.2	43
DS108	2.13 ± 0.81	76.5

Abbreviations: FP, fluorescence polarization; NRP1, neuropilin‐1; SPR, surface plasmon resonance.

**Figure 6:**
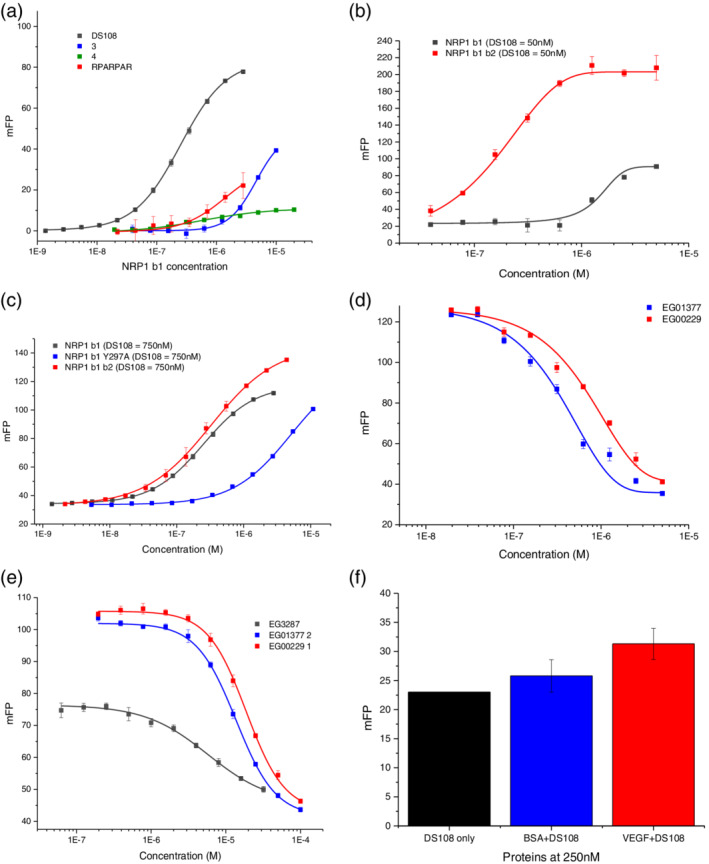
(a) Fluorescence polarization of NRP1‐B1 protein titrations against Flu‐tagged chemical probes, RPARPAR, **3**, **4**, and DS108. (b) Protein titrations of NRP1 b1, NRP1 b1b2 and NRP1 b1 mutant Y297A at 0.75 μM of DS108 (mutant suggests loss of affinity for DS108). (c) Representative fluorescence polarization competition experiments between the fluorescently tagged DS108 (0.75 μM) and EG01377, EG00229, and EG03287 for NRP1 b1 (7.5 μM). (d) Protein titrations of NRP1 b1 and NRP1 b1b2 at 50 μM of DS108. NRP1 b1b2 results in a better assay window than NRP1 b1 alone (e) Representative fluorescence polarization competition experiments between the fluorescently tagged DS108 (50 nM) and EG01377 and EG00229 for NRP1 b1b2 (0.3 μM). (f) Nonspecific binding of VEGF on the DS108 probe

With steady‐state equilibrium binding dissociation constant K_D_ values from SPR analysis in hand, fluorescently tagged RPARPAR, **3**, **4**, and DS108 were also evaluated for their utility as probes in FP experiments (Table 1). Based on this information and FP guidelines (Moerke, 2009) we assessed the dynamic FP window for the probes via a protein titration experiment (Figure 6, Table 2).

**Table 2:** FP binding properties of fluorescent probes to NRP1‐b1 protein

	FP			
5‐flu probe	Probe conc (μM)	NRP‐b1 conc (μM)	Dynamic window (mP)	NRP1 b1 K_D_ (μM)^a^
RPARPAR	7	2.81^b^	22.6	1.88 ± 1.4
(**3**)	1	10	30.7	4.61 ± 0.19
(**4**)	0.3	3	5.2	0.62 ± 0.098
DS108	0.75	7.5	77.8	0.248 ± 0.097

Abbreviations: FP, fluorescence polarization; NRP1, neuropilin‐1.

a K_D_ derived from the 50% signal maximum.

b Maximum NRP1‐b1 concentration attempted.

FP conditions should allow for ~50% of the probe being bound to the NRP1‐b1 protein, thus maximizing the potential readout window (Du, 2015; Lakowicz, 1983). Interestingly, the readout window between the “probe + NRP1‐b1” and “probe only” wells differed markedly for each probe (Table 2).

In our hands 5‐Flu‐RPARPAR probe showed poor affinity for NRP‐b1, as assessed by SPR, and this was coupled with only a modest dynamic window observed in FP (Tables 1 and 2).

Despite probe **4** demonstrating reasonable binding properties by SPR (K_D_ = 3.3 ± 0.2 μM), only a very small dynamic window could be obtained (Table 2; Figures 5a, and 6a). In contrast, probe **3** and DS108 showed low μM SPR binding properties, and this correlated with improved FP dynamic window readouts (Table 1). DS108 in particular appeared to possess a superior readout window, and this was confirmed by running full NRP1‐b1 titration curves for each probe (Figure 6a). Pleasingly, DS108 not only showed the greatest FP window but this probe was also able to elicit robust FP dynamic windows at significantly lower NRP1‐b1 protein concentrations than the literature FP probe, 5‐Flu‐RPARPAR, which is an important practical consideration for high throughput drug screening. As a check on the specificity of the probe we evaluated it in a protein titration experiment with NRP1 b1b2 and the NRP1 b1 Y297A mutated protein (Figure 6b). The probe showed very similar binding to both NRP1 b1 and the more complete receptor NRP1 b1b2. The NRP1 b1 Y297A mutant changes the structure of the binding domain and has been previously shown to reduce VEGF‐A binding (Herzog, Pellet‐Many, Britton, Hartzoulakis, & Zachary, 2011). DS108 showed markedly reduced binding to this mutated protein.

With a useful probe (DS108) and FP conditions now established, a competition experiment was performed. DS108 was first examined against the gold standard NRP1 antagonist EG00229 **1** and its analogue compound EG01377 **2**. Concentration‐dependent displacement of the probe was observed (Figure 6c). We also wanted to discover whether a structurally diverse competitor would displace this probe. We also employed the bicyclic disulfide bonded peptide, EG03287, which is derived from the C‐terminal domain of VEGF‐A165, as a competitor compound in this assay. Gratifyingly, concentration‐dependent competition of DS108 was also observed with EG03287 (Figure 6c) though we observed a reduced maximal signal. It is possible that the peptide ligand EG3287 is not able to fully displace the fluorescent probe. IC_50_ values were 20.47 ± 0.091, 16.84 ± 1.25, and 5.77 ± 0.87 μM EG00229, EG01377, and EG03287, respectively.

To check the relevance of this assay the inhibition constant (K_i_) was calculated. Using kinetic equations (Nikolovska‐Coleska et al., 2004), inhibition constants (K_i_ values) of 9.0, 7.39, and 2.47 μM were calculated for EG00229, EG01377, and EG03287, respectively, which were in agreement (within 1 log concentration unit) with competition and/or binding assays in the literature (Table 3) (Jarvis et al., 2010; Jia et al., 2006; Powell et al., 2018). In addition, the z‐factor for this assay was determined to be 0.90, suggesting its statistical robustness.

**Table 3:** Summary of fluorescence polarization (FP) competition experiment analyses and comparison to literature values

Compound	IC_50_ (μM)	Literature IC_50_ (μM)^a^	K_i_ (μM)^b^
EG00229	20.47 ± 0.091	8.0 (Jarvis et al., 2010)	9.0
EG01377	16.84 ± 1.25	1.32 (Powell et al., 2018)	7.39
EG03287	5.77 ± 0.87	1.20 (Jia et al., 2006)	2.47

*Note:* Z‐factor for this assay was 0.90.

a Literature values for NRP1 inhibition using bt‐VEGF_165_ binding, reference indicated.

b Ki values calculated using method in reference.

Final optimization of the assay was conducted by evaluating different buffers, and lower probe and DMSO concentrations. Then, 50 nM of probe concentration was chosen as it was still detectable by the plate reader without compromising the assay's accuracy. First, different concentrations of NRP1 b1 and b1b2 were titrated with 50 nM of probe in order to find out whether this low concentration was able to create a satisfactory assay window. As shown in Figure 6d NRP1 b1b2 domains created a good assay window. From the titration curve 300 nM of NRP1 b1b2 was chosen as it increased the initial signal by 65%. The optimal increase in signal is considered to lie between 50 and 80% as below 50% the assay window is poor and above 80% competition is visualized and the competition assay curve is expected to be a straight line. Secondly, a competition assay was performed using either EG00229 or EG01377. At 0.5% DMSO and using 10 mM of HEPES buffer the assay was able to generate a competition assay curve for both EG00229 and EG01377 (Figure 6e). HEPES buffer was found to be superior to PBS or PBS‐P. Miniaturization of the initial FP assay can be exploited for high throughput screening for NRP1 b1b2 inhibitors with expected Ki at the micromolar range. FP assay instruments can read a 384‐well plate at around 10 min and at the same time the miniaturized nature of the optimized protocol constitutes a cost effective alternative to the existing screening methods. Finally, the initial protocol was unable to generate a VEGF165 (natural ligand of NRP1 b1b2) competition assay curve. However, the optimized protocol revealed that the reason for this was nonspecific binding of DS108 to VEGF165, suggesting once more the sensitivity of the optimized protocol (Figure 6f).

## 5 CONCLUSION

We have identified DS108, a novel and convenient EG01377‐based fluorescent probe for evaluation of NRP1 binding, an important growth factor receptor that is implicated in the progression of various cancers. The probe was utilized as the ligand for a competitive FP binding assay. This probe could serve as a useful addition to the technical tools available for NRP1 study.

## CONFLICT OF INTEREST

None to report.

## AUTHOR CONTRIBUTIONS

E.D., D.S., and C.S. synthesized the compounds, E.D., V.N., Y.C., D.C., and F.M. did the biological assays. D.S. conceived the project. D.C., A.P., S.D., and D.S. wrote the paper.

## Supporting information


**Appendix** **S1**: Supporting information
