# Supplementary material for: Discovery of a novel fluorescent chemical probe suitable for evaluation of neuropilin‐1 binding of small molecules
Source: Drug Dev Res. 2020 Jan 20;81(4):491–500. doi: 10.1002/ddr.21641 (PMC7317715; doi:10.1002/ddr.21641)
Supplement: Supplementary file 1 — Appendix S1: Supporting information [file DDR-81-491-s001.docx]

Supplementary information LCMS of DS108

DS108
